# 内源T细胞的旁观效应在CD19 CAR-T细胞治疗后淋巴瘤不同阶段的表型及功能变化

**DOI:** 10.3760/cma.j.cn121090-20260129-00066

**Published:** 2026-08

**Authors:** 芬 颜, 东海 唐, 雯雯 李, 颖 顾, 春晓 任, 威 桑, 恺 赵

**Affiliations:** 1 徐州医科大学附属医院血液科，徐州 221002 Department of Hematology, the Affiliated Hospital of Xuzhou Medical University, Xuzhou 221002, China; 2 徐州医科大学血液病研究所，徐州 221002 Institute of Hematology, Xuzhou Medical University, Xuzhou 221002, China; 3 淮海医学科技创新与公共服务中心，徐州 221005 Huaihai Medical Science and Technology Innovation and Public Service Center, Xuzhou 221005, China

**Keywords:** 淋巴瘤，B细胞, 内源T细胞, CD19 CAR-T细胞, 肿瘤微环境, Lymphoma, B-cell, Endogenous T cell, CD19 CAR-T cell, tumor microenvironment

## Abstract

**目的:**

探索肿瘤微环境中内源T细胞的旁观效应及其在CD19嵌合抗原受体T（CAR-T）细胞治疗B细胞淋巴瘤缓解及复发期中的作用及表型变化。

**方法:**

在徐州医科大学血液病研究所利用BALB/c小鼠构建CD19 CAR-T细胞治疗CD19^−^及CD19^+^A20淋巴瘤的体外和小鼠模型，其中体外实验根据共培养的细胞类型分为活化T细胞组（3只）和CD19 CAR-T细胞组（3只），CD19^−^A20淋巴瘤小鼠模型根据治疗的细胞类型分为活化T细胞治疗组（5只）和CD19 CAR-T细胞治疗组（5只）；而CD19^+^A20淋巴瘤小鼠模型则依据治疗的细胞类型或疾病状态分为活化T细胞治疗组（3～8只）、CD19 CAR-T细胞治疗组（14只），缓解组（临床评分≤1分）和复发组（临床评分>1分），评估治疗效果并通过流式细胞术对不同疾病状态下内源T细胞的亚群、活化状态、细胞因子分泌情况进行检测。

**结果:**

体外实验表明，与活化T细胞组相比，CD19 CAR-T细胞对CD19^−^A20细胞无特异性杀伤作用［存活率：（40.83±1.70）％对（39.07±1.40）％，*P*＝0.238］；但在免疫健全的小鼠体内，相比于活化T细胞治疗组，CD19 CAR-T细胞治疗后CD19^−^A20皮下肿瘤的重量减轻［（0.14±0.10）g对（0.81±0.24）g，*P*＝0.002］、体积显著缩小［（335.40±130.20）mm^3^对（542.00±302.40）mm^3^，*P*＝0.004］，且内源CD3^+^T细胞浸润比例更高［（36.34±6.53）％对（9.49±9.42）％，*P*＝0.001］，CD69^+^CD8^+^T细胞比例更高［（21.38±4.85）％对（3.15±1.16）％，*P*＝0.001］，表达耗竭相关分子PD-1及TIGIT细胞比例呈下降趋势（*P*值均>0.05）。在CD19^+^A20淋巴瘤缓解和复发小鼠模型中，CD19 CAR-T细胞治疗复发后脾脏（SP）及骨髓（BM）中内源CD8^+^T细胞比例较缓解期呈下降趋势（*P*值均>0.05），CD69表达强度［平均荧光强度（MFI）］降低［CD4^+^T细胞：BM：733.20±274.40对1 093.00±131.20，*P*＝0.013；SP：290.50±142.40对478.70±63.94，*P*＝0.018；CD8^+^T细胞：BM：1 223.00±391.50对1 892.00±540.40，*P*＝0.041；SP：342.30±258.10对500.00±113.90，*P*＝0.325］，细胞因子IFN-γ、TNF-α、GM-CSF、颗粒酶B的分泌水平均下降（*P*值均<0.05）；复发期效应T细胞比例均明显减少，而初始T细胞及记忆T细胞比例明显增加（*P*值均<0.05）。

**结论:**

CD19 CAR-T细胞体内的特异性杀伤可激活内源T细胞产生旁观效应，从而非特异性抑制淋巴瘤生长，但疾病复发后内源T细胞杀伤功能逐渐减弱并向静息及记忆性表型转化。

## Abstract (en)

**Objective:**

To investigate the bystander effect of endogenous T cells within the tumor microenvironment, as well as their roles and phenotypic changes during remission and relapse in mice with B-cell lymphoma receiving CD19-targeted chimeric antigen receptor T（CAR-T）cell therapy.

**Methods:**

At the Institute of Hematology, Xuzhou Medical University, in vitro experiments and mouse models were established using BALB/c mice to evaluate the efficacy of CD19 CAR-T cells in treating CD19^−^ and CD19^+^A20 lymphomas. In the in vitro experiments, the mice were categorized into the following groups based on the type of co-cultured cells: activated T cell（*n*＝3）and CD19-targeted CAR-T cell（*n*＝3）groups. In the CD19^−^ A20 lymphoma mouse model, the mice were assigned to the activated T-cell（*n*＝5）and CD19 CAR-T cell treatment（*n*＝5）groups based on the type of cells used for treatment. For the CD19^+^ A20 lymphoma mouse model, the mice were divided into the activated T cell treatment（*n*＝3–8）, CD19 CAR-T cell treatment（*n*＝14）, remission（clinical score ≤ 1）, and relapse（clinical score >1）groups. Therapeutic efficacy was evaluated, and flow cytometry was used to analyze the subsets, activation status, and cytokine secretion profiles of endogenous T cells at different disease states.

**Results:**

In vitro experiments demonstrated that, compared with the activated T-cell group, CD19 CAR-T cells exerted no specific cytotoxic effect on CD19^−^ A20 cells［survival rates:（40.83±1.70）％*vs*（39.07±1.40）％, *P*＝0.238］. However, in immunocompetent mice, compared with the activated T-cell treatment, CD19-targeted CAR-T cell therapy resulted in reduced weight［（0.14±0.10）*vs*（0.81±0.24）g, *P*＝0.002］and significantly diminished subcutaneous CD19^−^ A20 tumor volume［（335.40±130.20）*vs*（542.00±302.40）mm^3^, *P*＝0.004］, accompanied by increased infiltration of endogenous CD3^+^ T cells［（36.34±6.53）％ *vs*（9.49±9.42）％, *P*＝0.001］. CD8^+^ T cells exhibited increased CD69 expression［（21.38±4.85）％ *vs*（3.15±1.16）％, *P*＝0.001］and a trend toward decreased expression of exhaustion-associated molecules PD-1 and TIGIT（all *P* value >0.05）. In the mouse model of CD19^+^ A20 lymphoma remission and relapse, upon disease relapse, the proportion of endogenous CD8^+^ T cells in the spleen（SP）and bone marrow（BM）showed a downward trend during disease relapse compared with that during the remission period, the CD69 expression intensity decreased（CD4^+^ T cell: BM: 733.20±274.40 *vs* 1 093.00±131.20, *P*＝0.013; SP: 290.50±142.40 *vs* 478.70±63.94, *P*＝0.018; CD8^+^ T cell: BM: 1 223.00±391.50 *vs* 1 892.00±540.40, *P*＝0.041; SP: 342.30±258.10 *vs* 500.00±113.90, *P*＝0.325）and the secretion levels of cytokines IFN-γ, TNF-α, GM-CSF, and granzyme B were all diminished（all *P* value <0.05）. During relapse, the proportion of effector T cell（Teff）cells decreased significantly, whereas the proportions of naive T cell（Tn）cells and memory T cell（Tm）cells increased significantly（all *P* value <0.05）.

**Conclusion:**

The specific cytotoxic activity of CD19-targeted CAR-T cells in vivo can activate endogenous T cells and induce a bystander effect, which non-specifically inhibits lymphoma growth. However, following disease relapse, the cytotoxic function of endogenous T cells gradually diminishes and shifts toward resting and memory phenotypes.

CD19嵌合抗原受体T（CAR-T）细胞治疗复发难治性B细胞淋巴瘤已取得显著疗效，但相当一部分接受CD19 CAR-T细胞治疗有效的患者疾病进展或复发[1]–[3]。肿瘤微环境（TME）作为CAR-T细胞治疗长期缓解的影响因素，其免疫响应及调控，可作用于治疗全程并影响患者预后[1],[4]–[6]。

旁观效应T细胞是肿瘤浸润淋巴细胞中的主要亚群，以不依赖于T细胞受体而依赖于细胞因子的方式被激活，具有CD8、CD160、NKG2D和CCL5等标志物，属于内源T细胞的精细亚群[7]–[8]。Kaminski等[8]报道，靶向CD20的CAR-T细胞可激活旁观CD8^+^CAR^neg^T细胞亚群，从而促进肿瘤溶解。有研究发现，CD19 CAR-T细胞可协同内源T细胞分泌的γ干扰素（IFN-γ）下调VISTA表达，增强内源T细胞的细胞活性并促其向记忆性T细胞转变，从而有效杀伤肿瘤细胞的同时延长CAR-T细胞治疗的持续时间，且其亚群的组成及功能变化与临床反应相关[9]–[10]。以上提示在TME中存在内源T细胞的旁观效应，即内源T细胞独立于同源抗原识别而被激活，其特征是活化标志物上调、效应功能的获得并伴有细胞亚群构成的改变，与CAR-T细胞治疗疗效密切相关。因此，动态监测TME中内源T细胞的功能转化，增强CAR-T细胞治疗后的旁观效应，是增强疗效、减少复发的潜在重要策略。

本研究利用CAR-T细胞治疗的淋巴瘤小鼠模型，分析不同疾病状态下内源T细胞的组成及功能变化，旨在阐明内源T细胞的旁观效应在CAR-T细胞治疗疗效维持与复发抵抗中的变化，为开发“特异性杀伤”联合“非特异性旁观杀伤”的新型治疗策略提供实验依据。

## 材料与方法

1. 细胞系与实验动物：A20细胞系（小鼠B细胞淋巴瘤细胞）由徐州医科大学血液病研究所潘彬教授赠送，并由本实验室自行保存，使用添加10％胎牛血清的RPMI 1640培养基进行培养。雌性BALB/c小鼠（6～8周，20 g，无特殊病原体级别）购自北京维通利华实验动物有限公司，饲养于具备独立通气笼具（IVC）系统的徐州医科大学血液病研究所动物中心。根据实验分组，每组小鼠3～14只不等。实验结束后以1.25％三溴乙醇溶液（300 µl，南京爱贝生物科技有限公司）麻醉后脱颈处死小鼠。本研究的所有实验程序均通过徐州医科大学实验动物伦理委员会审批（批件号：202412T008），所有实验均重复3次。

2. CD19 CAR-T细胞的制备：与本课题组先前报道的方法相同[11]，简单描述如下，使用EasySep™ Mouse T Cell Isolation Kit（加拿大StemCell Technologies公司）从BALB/c小鼠脾中分离纯化CD3^+^ T细胞，接种于预先包被有抗CD3单克隆抗体（2 mg/ml，美国BioLegend公司）和可溶性抗CD28单克隆抗体（2 µg/ml，美国BioLegend公司）的24孔板中激活36 h。以感染复数（MOI）＝1，向活化的T细胞中加入含有逆转录病毒颗粒（由携带CAR基因的逆转录病毒载体pMX-2A-EGFP-Flag包装）的细胞上清液，转染72 h后，通过流式细胞术检测GFP^+^ CAR-T细胞的百分比，转染效率达（32.27±7.56）％。

3. CD19^−^A20细胞获取及体外共培养体系建立：CD19^+^A20细胞连续传代，获取的标志物缺失的细胞定义为CD19^−^A20细胞。用CellTrace™ Far Red（美国invitrogen公司）对CD19^−^A20细胞进行标记，将3×10^5^个活化T细胞或CD19 CAR-T细胞与CD19^+^A20细胞或CD19^−^A20细胞按照效靶比1:1共培养24 h后，利用流式细胞术进行检测。先以前向角散射光（FSC）/侧向角散射光（SSC）设门单个活细胞，再以A20^+^Far Red^+^设门圈定CD19^−^A20细胞，以A20^+^CD19^+^设门圈定CD19^+^A20细胞。

4. 小鼠模型的建立与评估：所有小鼠通过耳标进行唯一标识，并按随机数字表法随机分配至各实验组。① CD19^−^A20皮下淋巴瘤模型的构建：将Matrigel™基质胶与PBS溶液按1:1比例混合后重悬CD19^−^A20细胞至1×10^7^/ml，随后将200 µl细胞悬液皮下注入经⁶⁰Co γ射线照射，单次吸收剂量为3.5 Gy的BALB/c小鼠右腹部。1周后通过尾静脉给予1×10^6^个活化T细胞或CD19 CAR-T细胞治疗，治疗后每2天测量肿瘤的长（a）、短径（b），体积（V）计算公式为V＝ab^2^/2。② CD19^+^A20淋巴瘤缓解和复发小鼠模型构建：将1×10^6^个A20细胞通过尾静脉注射至经⁶⁰Co γ射线照射，单次吸收剂量为3.5 Gy的BALB/c小鼠体内。3 d后再次通过小鼠尾静脉输注2×10^5^个活化T细胞或CD19 CAR-T细胞治疗。监测小鼠的存活情况，每2天测量1次小鼠重量。根据本中心前期研究的模型评分标准，综合小鼠重量变化、活动度和瘫痪程度对其进行临床症状评分[6]。临床评分≤1分为缓解期，而临床评分>1分为疾病复发期。总生存（OS）期定义为回输CAR-T细胞当天至任何原因导致小鼠死亡的时间；无进展生存（PFS）期定义为回输CAR-T细胞当天至小鼠临床评分>1分或死亡的时间。

5. 流式细胞术检测及抗体：经研磨和红细胞裂解液处理，从小鼠皮下肿瘤、脾（SP）及骨髓（BM）中制备单个核细胞悬液。PBS洗涤后，使用单克隆抗体混合液对各组织来源的细胞进行染色。所使用的抗体及来源：CD3-BV650（货号：100204）、CD19-PE/Dazzle™ 594（货号：115553）、CD4-AF700（货号：100536）、CD8-APC（货号：100712）、CD8-APC-CY7（货号：100714）、CD69-BV510（货号：104532）、CD62L-PB（货号：104424）、程序性死亡受体1（PD-1）-PE-CY7（货号：109110）、T细胞免疫球蛋白ITIM结构域（TIGIT）-PE（货号：142104）、IFN-γ-PE-CY7（货号：505826）、肿瘤坏死因子-α（TNF-α）-APC（货号：506308）、粒细胞-巨噬细胞集落刺激因子（GM-CSF）-PE（货号：505405）购自美国BioLegend公司；CD44-APC/eFlour780（货号：2611857）购自美国Invitrogen公司；颗粒酶B（GzmB）-eFlour450（货号：E15759-103）购自武汉伊莱瑞特生物科技股份有限公司。使用BD LSRFortessa流式细胞仪（RRID：SCR_019601）进行检测、FlowJo（v10.6.2）进行流式细胞术数据分析。以FSC/SSC设门单个活细胞，根据CD19表达分为CD19⁺A20和CD19⁻细胞群。在CD19⁻细胞中圈出CD3⁺GFP⁺的CAR-T细胞和CD3⁺GFP⁻的内源T细胞，并分析CD4⁺和CD8⁺內源T细胞及其表型（CD69、CD44、CD62L、PD-1和TIGIT）和功能（IFN-γ、TNF-α、GM-CSF和GzmB）。

6. 统计学处理：Graphpad prism 9进行数据的统计及作图。计量资料以*x*±*s*表示，两组间的比较采用*t*检验，多组间比较采用单因素或多因素方差分析，生存分析采用Log-rank检验，生存时间以*M*（95％ *CI*）表示。*P*<0.05为差异有统计学意义。

## 结果

1. CD19 CAR-T细胞对CD19^−^A20淋巴瘤的旁观杀伤效应：体外共培养体系中，同活化T细胞组相比，CD19 CAR-T细胞可杀伤近100％的CD19^+^A20细胞［细胞存活率：（0.09±0.15）％对（29.83±1.79）％，*P*<0.001］，而对CD19^−^A20生存无影响［（40.83±1.70）％对（39.07±1.40）％，*P*＝0.238］，提示CD19靶点抗原的特异性杀伤作用。然而在体内实验中，与接受活化T细胞治疗的CD19^−^A20淋巴瘤小鼠相比，接受CD19 CAR-T细胞治疗的小鼠肿瘤重量降低［（0.14±0.10）g对（0.81±0.24）g，*P*＝0.002］且体积缩小［（335.40±130.20）mm^3^对（542.00±302.40）mm^3^，*P*＝0.004］（图1）。流式细胞术分析显示（表1），CD19 CAR-T细胞治疗组小鼠肿瘤浸润的CD3^+^ T细胞的比例高于活化T细胞治疗组［（36.34±6.53）％对（9.49±9.42）％，*P*＝0.001］。尽管内源CD4^+^T细胞中活化分子CD69以及耗竭相关分子PD-1、TIGIT的比例无明显变化（*P*值均>0.05），但相比于活化T细胞组，CD19 CAR-T细胞治疗后具备直接杀伤能力的内源CD8^+^T细胞中，CD69比例显著上调［（21.38±4.85）％对（3.15±1.16）％，*P*<0.001］，PD-1［（16.88±3.94）％对（23.81±14.02）％，*P*＝0.340］及TIGIT［（8.32±3.18）％对（11.58±8.18）％，*P*＝0.443］均呈下降趋势。上述结果提示，CD19 CAR-T细胞可能通过激活内源T细胞的旁观效应实现对CD19^−^A20细胞的非特异性清除，抑制肿瘤生长。

**图1:**
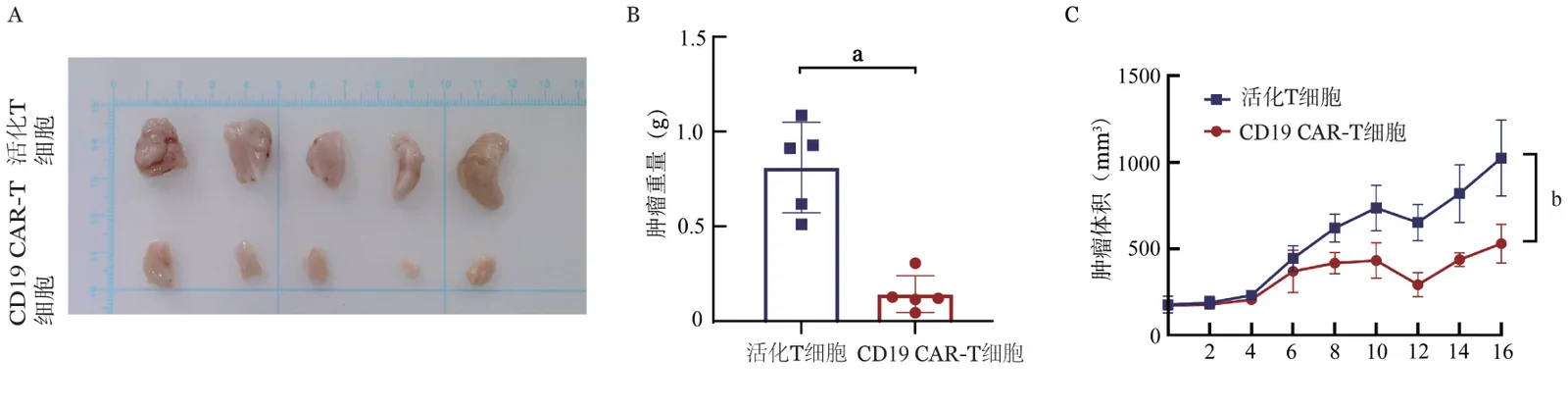
小鼠模型验证CD19 CAR-T细胞通过旁观效应杀伤CD19^−^A20细胞 **A** CD19^−^A20淋巴瘤经活化T细胞（5只）及CD19 CAR-T细胞（5只）治疗后的小鼠皮下肿瘤情况；**B** 活化T细胞及CD19 CAR-T细胞治疗CD19^−^A20淋巴瘤时肿瘤重量的对比情况；**C** 活化T细胞及CD19 CAR-T细胞治疗CD19^−^A20淋巴瘤时肿瘤体积的变化情况 **注** CAR-T细胞：嵌合抗原受体T细胞；a：*P*<0.001；b：*P*<0.01

**表1:** 活化T细胞与CD19 CAR-T细胞治疗CD19^−^A20淋巴瘤后T细胞及其亚群活化与耗竭分子细胞比较（％，*x*±*s*）

细胞比例类型	活化T细胞治疗组（5只）	CD19 CAR-T细胞治疗组（5只）	*t*值	*P*值
CD3^+^细胞比例	9.49±9.42	36.34±6.53	5.24	0.001
CD4^+^CD69^+^T细胞比例	4.76±3.13	26.58±19.54	2.47	0.066
CD8^+^CD69^+^T细胞比例	3.15±1.16	21.38±4.85	8.17	<0.001
CD4^+^PD-1^+^T细胞比例	50.18±17.55	46.32±17.07	0.35	0.733
CD8^+^PD-1^+^T细胞比例	23.81±14.02	16.88±3.94	1.06	0.340
CD4^+^TIGIT^+^T细胞比例	27.38±14.31	27.45±16.15	<0.01	0.995
CD8^+^ TIGIT ^+^T细胞比例	11.58±8.18	8.32±3.18	0.83	0.443

**注** CAR-T细胞：嵌合抗原受体T细胞；PD-1：程序性死亡受体1；TIGIT：T细胞免疫球蛋白ITIM结构域

2. CD19 CAR-T细胞对小鼠淋巴瘤的疗效：构建CD19^+^A20的免疫健全小鼠淋巴瘤模型，分析CD19 CAR-T细胞治疗淋巴瘤不同疾病阶段的内源T细胞变化情况。与活性T细胞治疗组（8只）相比，CD19 CAR-T细胞治疗（14只）的小鼠OS期［34.0（95％ *CI*：17.0～65.0）d对11.0（95％ *CI*：无法估计）d，*P*<0.001］和PFS期［31.5（95％ *CI*：15.0～63.0）d对9.0（95％ *CI*：9.0～10.0）d，*P*<0.001］显著延长。CD19 CAR-T细胞治疗后第15天，92.9％（13/14）的小鼠处于缓解期，且相比于活化T细胞治疗组SP及BM中CD19^+^肿瘤细胞比例明显降低［SP：（4.60±6.96）％对（27.69±17.29）％，*P*＝0.102；BM：（4.26±5.63）％对（52.63±11.80）％，*P*<0.001］。治疗时间延长到第45天时，仅有57.1％（8/14）的小鼠处于缓解期，42.9％的小鼠（6/14）临床评分>1分，与缓解状态相比，SP和BM中CD19^+^肿瘤细胞比例均较高［SP：（22.12±22.13）％对（4.60±6.96）％，*P*＝0.246；BM：（7.74±9.82）％对（4.26±5.63）％，*P*＝0.801］（表2）。上述模型展示CD19 CAR-T细胞治疗后缓解和复发的疾病状态，为后续研究不同疾病阶段内源T细胞的免疫学特性提供了可信数据。

**表2:** CD19 CAR-T细胞治疗后CD19^+^ A20小鼠不同疾病阶段免疫细胞亚群及功能状态比较（％，*x*±*s*）

类型	活化T细胞治疗组（6只）	缓解组（6只）	复发组（6只）	统计量1（*q*值）^a^	*P*1值^a^	统计量2（*q*值）^b^	*P*2值^b^	统计量3（*q*值）^c^	*P*3值^c^
脾									
CD19^+^肿瘤细胞比例	27.69±17.29	4.60±6.96	22.12±22.13	3.14	0.102	0.79	0.842	2.38	0.246
CD4^+^T细胞比例	88.25±5.19	89.22±1.83	93.28±4.28	0.59	0.910	3.06	0.110	2.48	0.220
CD8^+^T细胞比例	11.63±5.17	10.64±1.83	6.56±3.94	0.62	0.901	3.19	0.094	2.57	0.198
CD4^+^T细胞CD69 MFI（*x*±*s*）	485.20±89.87	478.70±63.94	290.50±142.40	0.15	0.994	4.59	0.014	4.43	0.018
CD8^+^T细胞CD69 MFI（*x*±*s*）	575.70±146.90	500.00±113.90	342.30±258.10	1.01	0.759	3.11	0.103	2.10	0.325
CD4^+^T细胞Teff比例	36.57±6.94	47.98±10.54	27.27±7.24	3.33	0.079	2.71	0.168	6.04	0.002
CD8^+^T细胞Teff比例	32.02±13.11	44.98±17.76	25.93±27.23	1.57	0.523	0.74	0.863	2.31	0.264
CD4^+^T细胞Tm比例	6.24±3.11	4.51±1.83	6.77±3.82	1.39	0.597	0.43	0.950	1.83	0.421
CD8^+^T细胞Tm比例	10.63±6.99	9.26±6.09	13.61±7.45	0.49	0.937	1.07	0.736	1.55	0.530
CD4^+^T细胞Tn比例	22.12±9.72	12.39±7.73	47.35±16.53	2.00	0.360	5.18	0.006	7.17	<0.001
CD8^+^T细胞Tn比例	18.95±12.34	9.97±8.21	47.07±24.82	1.32	0.630	4.13	0.027	5.44	0.004
IFN-γ^+^CD4^+^T细胞比例	3.04±1.03	9.36±3.27	2.01±1.58	7.11	<0.001	1.16	0.696	8.27	<0.001
IFN-γ^+^CD8^+^T细胞比例	27.38±13.40	34.72±7.44	5.39±3.09	1.99	0.362	5.97	0.002	7.96	<0.001
TNF-α^+^CD4^+^T细胞比例	8.12±4.15	17.15±7.59	8.37±4.00	4.02	0.031	0.11	0.997	3.91	0.036
TNF-α^+^CD8^+^T细胞比例	12.36±8.24	16.90±8.57	4.51±2.55	1.58	0.518	2.74	0.163	4.32	0.021
GzmB^+^CD4^+^T细胞比例	6.23±1.92	6.88±2.79	1.18±0.44	0.80	0.830	6.28	0.001	7.09	<0.001
GzmB^+^CD8^+^T细胞比例	7.99±2.75	6.28±3.91	1.50±0.87	1.49	0.556	5.67	0.003	4.18	0.025
GM-CSF^+^CD4^+^T细胞比例	15.81±5.28	13.18±4.84	0.48±0.29	1.56	0.528	9.07	<0.001	7.52	<0.001
GM-CSF^+^CD8^+^T细胞比例	18.91±6.16	12.75±6.19	0.21±0.12	2.99	0.120	9.08	<0.001	6.09	0.002
骨髓									
CD19^+^肿瘤细胞比例	52.63±11.80	4.26±5.63	7.74±9.82	12.55	<0.001	11.65	<0.001	0.90	0.801
CD4^+^T细胞比例	61.00±7.42	74.67±10.39	84.37±16.00	2.83	0.146	4.84	0.010	2.01	0.355
CD8^+^T细胞比例	38.88±7.41	25.15±10.41	15.45±15.95	2.85	0.143	4.87	0.010	2.01	0.354
CD4^+^T细胞CD69 MFI（*x*±*s*）	1 099.00±122.70	1 093.00±131.20	733.20±274.40	0.07	0.999	4.73	0.012	4.66	0.013
CD8^+^T细胞CD69 MFI（*x*±*s*）	1 669.00±331.20	1 892.00±540.40	1 223.00±391.50	1.27	0.650	2.54	0.204	3.81	0.041
CD4^+^T细胞Teff比例	54.73±7.33	75.78±8.44	56.02±5.11	7.27	<0.001	0.44	0.948	6.82	<0.001
CD8^+^T细胞Teff比例	90.52±1.15	87.93±3.85	76.85±8.16	1.21	0.678	6.37	0.001	5.17	0.006
CD4^+^T细胞Tm比例	3.02±1.01	4.28±1.55	12.11±5.60	0.91	0.799	6.55	<0.001	5.64	0.003
CD8^+^T细胞Tm比例	3.88±1.33	9.67±3.24	16.95±4.57	4.26	0.022	9.62	<0.001	5.36	0.005
CD4^+^T细胞Tn比例	6.11±1.99	1.12±0.49	8.66±5.77	3.46	0.066	1.77	0.444	5.23	0.006
CD8^+^T细胞Tn比例	0.56±0.21	0.22±0.09	2.74±1.13	1.23	0.666	8.03	<0.001	9.26	<0.001
IFN-γ^+^CD4^+^T细胞比例	13.89±5.06	14.64±6.07	11.90±9.58	0.27	0.980	0.65	0.891	0.89	0.806
IFN-γ^+^CD8^+^T细胞比例	53.08±8.12	52.17±20.71	25.78±9.34	0.15	0.993	4.12	0.030	3.99	0.036
TNF-α^+^CD4^+^T细胞比例	13.09±4.24	12.66±6.98	14.35±4.85	0.19	0.990	0.49	0.935	0.66	0.886
TNF-α^+^CD8^+^T细胞比例	23.57±6.29	30.22±11.08	9.24±4.60	1.99	0.368	3.83	0.045	5.60	0.004
GzmB^+^CD4^+^T细胞比例	0.39±0.14	4.35±7.17	0.23±0.11	2.18	0.304	0.08	0.998	2.03	0.353
GzmB^+^CD8^+^T细胞达比例	0.46±0.34	0.54±0.84	0.14±0.20	0.35	0.967	1.22	0.671	1.54	0.539
GM-CSF^+^CD4^+^T细胞比例	21.39±8.62	14.75±6.78	1.05±0.62	2.39	0.247	6.54	0.001	4.41	0.021
GM-CSF^+^CD8^+^T细胞比例	23.03±9.96	20.25±10.85	1.98±2.31	0.74	0.861	5.01	0.009	4.35	0.022

**注** CAR-T细胞：嵌合抗原受体T细胞；MFI：平均荧光强度；Teff：效应T细胞；Tm：记忆T细胞；Tn：初始T细胞；IFN-γ：干扰素-γ；TNF-α：肿瘤坏死因子-α；GzmB：颗粒酶B；GM-CSF：粒细胞-巨噬细胞集落刺激因子；缓解组：临床评分≤1分；复发组：临床评分>1分；^a^：活化T细胞治疗组与缓解组比较；^b^：活性T细胞治疗组与复发组比较；^c^：缓解组与复发组比较

3. 缓解和复发期内源T细胞活性及亚群的变化：利用流式细胞术对CD19 CAR-T细胞治疗后不同时期的内源T细胞进行分析（表2），与缓解期相比，复发时SP和BM中CD8^+^T细胞比例有下降趋势（*P*值均>0.05）。进一步检测显示CD4^+^T（SP：290.50±142.40对478.70±63.94，*P*＝0.018；BM：733.20±274.40对1 093.00±131.20，*P*＝0.013）及CD8^+^T（SP：342.30±258.10对500.00±113.90，*P*＝0.325；BM：1 223.00±391.50对1 892.00±540.40，*P*＝0.041）细胞表面活化分子CD69的平均荧光强度（MFI）减弱，提示复发阶段内源T细胞的整体活化水平降低。为了进一步揭示内源T细胞功能亚群的变化，将内源T细胞分为效应T细胞（Teff）（CD44^+^CD62L^−^）、记忆T细胞（Tm）（CD44^+^CD62L^+^）与初始T细胞（Tn）（CD44^−^CD62L^+^）。如表2所示，与缓解期相比，复发期具有细胞毒性功能的Teff比例在CD4^+^T细胞和CD8^+^T细胞中均有不同程度的减少；而Tm亚群和Tn亚群的比例在CD4^+^T细胞和CD8^+^T细胞中则增加（*P*值均<0.05）。以上结果提示，在CD19 CAR-T细胞治疗后复发阶段，内源T细胞不仅数量组成发生变化，其功能分化状态也向记忆表型和初始表型偏移。

4. 缓解和复发期内源T细胞功能差异：通过对内源T细胞分泌细胞因子比例进行检测以评估功能，如表2所示，与CD19 CAR-T细胞治疗后缓解期相比，复发期SP中CD4^+^及CD8^+^T细胞分泌促炎细胞因子IFN-γ、TNF-α、GM-CSF及T细胞杀伤关键分子GzmB的细胞比例均明显降低（*P*值均<0.05），提示复发期SP中的T细胞的效应功能整体减弱。在BM中CD8^+^T细胞分泌细胞因子的细胞比例变化与SP一致，IFN-γ、TNF-α、GM-CSF的细胞比例均明显下降（*P*值均<0.05），分泌GzmB的细胞比例有下降趋势［（0.14±0.20）％对（0.54±0.84）％，*P*＝0.539］；CD4⁺T细胞中分泌GM-CSF的细胞比例降低［（1.05±0.62）％对（14.75±6.78）％，*P*＝0.021］，而IFN-γ、TNF-α和GzmB的细胞比例差异无统计学意义（*P*值均>0.05）。以上结果提示，在CD19 CAR-T细胞治疗后缓解和复发阶段，不同TME中T细胞的功能抑制程度存在差异，尤其是具有杀伤功能的CD8^+^ T细胞。

## 讨论

27％的患者在接受axi-cel细胞疗法（自体CD19 CAR-T细胞治疗）后出现了CD19阴性复发[12]，提示靶抗原阴性复发是CAR-T细胞治疗后原发病复发的机制之一。而包括CD20或CD22靶点的CAR-T细胞治疗、放疗或化疗、异基因造血干细胞移植在内的CD19阴性复发后的挽救治疗预后较差[13]。本研究中，CD19 CAR-T细胞在体外实验中对CD19^−^A20细胞并无杀伤作用；但在免疫健全小鼠体内，CD19 CAR-T细胞对CD19阴性肿瘤仍存在非特异性杀伤。因此推测CD19 CAR-T细胞特异性杀伤CD19^+^B细胞，从而促进CAR-T细胞的扩增及细胞因子的产生，进而激活旁观T细胞非特异性杀伤CD19^−^肿瘤细胞。明确CD19 CAR-T细胞治疗过程中旁观免疫细胞的变化，或许可为靶抗原阴性复发的治疗提供新思路。

本研究发现，缓解期小鼠体内内源CD4^+^和CD8^+^T细胞均表现出较强的活化状态，其活化标志物CD69的表达水平高于复发期小鼠，且分泌细胞因子（IFN-γ、TNF-α、GM-CSF）以及细胞毒性介质GzmB的能力也明显强于复发期。与前期研究结果一致，在A20淋巴瘤小鼠模型中，CAR-T细胞分泌的IFN-γ可下调内源T细胞上VISTA表达，促进内源T细胞激活[9]。以上提示在CD19 CAR-T细胞治疗后，内源T细胞的功能处于高度激活及有效的抗肿瘤状态，其可能协同CAR-T细胞清除残留的肿瘤细胞，并可能通过“抗原扩散”机制形成更强的免疫保护，而治疗后期CAR-T细胞自然耗竭且内源T细胞协同抗肿瘤能力下降，最终导致疾病复发[14]–[16]。此外，我们观察到缓解期Teff比例显著升高与其杀伤功能相一致，复发期Teff比例下降，Tm和Tn比例相对增加。这种亚群组成变化可能具有双重不利影响：一方面，内源Teff比例的减少可导致杀伤肿瘤的能力不足；另一方面，较高比例的Tm和Tn细胞处于静息状态，降低了免疫系统克隆多样性以及对新表位应答的潜能，削弱了免疫监视的持续性和适应性[17]–[19]，提示复发不仅是效应功能的缺失，更可能是免疫储备的耗竭。调节性T细胞（Treg）、髓源性抑制细胞（MDSC）、肿瘤相关巨噬细胞（TAM）等抑制性免疫细胞可导致CAR-T细胞治疗过程中内源T细胞的功能抑制[20]。本中心前期研究也证实，CAR-T细胞治疗后淋巴瘤复发时，小鼠皮下肿瘤、SP、BM及肝脏中TAM向促肿瘤的M2型极化[5]。另有研究表明，限制MDSC向肿瘤微环境中募集、清除TAM等可有效提高内源CD8^+^T细胞的存活率，并发挥显著的抗肿瘤作用[21]–[22]。提示疾病复发时内源T细胞功能的抑制，与Treg、MDSC和TAM等抑制性细胞的调控密切相关。

目前，已有临床策略尝试利用和调控内源T细胞的旁观效应改善治疗效果。如在CD19 CAR-T细胞治疗后缓解期，使用免疫检查点抑制剂抗PD-1维持内源T细胞的活化与功能状态[23]；利用病原肽激活TME中的旁观T细胞，克服免疫检查点耐药[24]。此外，CAR-T细胞治疗前常规使用的淋巴细胞清除方案可引起直接且长期的不良反应，包括不可逆的细胞毒性损伤、BM功能抑制等[25]。在联合利用内源T细胞旁观效应的治疗策略中，通过适当降低淋巴细胞清除方案的强度，可以在保证疗效的同时减少不良反应。这些干预手段在初步临床观察中已显示出良好的治疗前景，进一步印证了内源T细胞的旁观效应在增强细胞免疫应答、克服肿瘤治疗耐药、减少不良反应等方面具有重要潜力与显著优势。

总之，内源T细胞在CD19 CAR-T细胞治疗B细胞淋巴瘤的缓解与复发过程中扮演关键角色，激活内源T细胞的旁观效应是增强CAR-T细胞疗效的重要助力。然而，本研究主要基于荷瘤动物模型进行实验，尚未通过临床患者样本进行系统性验证，结论的临床适用性与普适性仍有待进一步验证；另外，本研究尚未在机制研究层面明确旁观效应所依赖的关键信号轴，相关分子机制仍需深入挖掘。基于本研究结果，未来可从单独改造CAR-T细胞转向协同激活内源T细胞，通过联合免疫检查点抑制剂或集落刺激因子1受体（CSF-1R）抑制剂解除免疫抑制、细胞因子装甲型CAR-T细胞提供免疫支持以及联合病原肽激活内源T细胞的免疫应答等多模式干预手段，同步增强CAR-T细胞特异性杀伤与内源T细胞的非特异性旁观效应，或许可成为淋巴瘤患者延长缓解期、减少复发的新策略。
